# Transcranial focused ultrasound-induced blood‒brain barrier opening in mice without shaving hairs

**DOI:** 10.1038/s41598-023-40598-4

**Published:** 2023-08-19

**Authors:** Lu Xu, Yan Gong, Chih-Yen Chien, Eric Leuthardt, Hong Chen

**Affiliations:** 1 Department of Biomedical Engineering, Washington University in St. Louis, Saint Louis, MO 63130 USA; 2 Department of Neurosurgery, Washington University School of Medicine, Saint Louis, MO 63110 USA; 3 Center for Innovation in Neuroscience and Technology, Washington University School of Medicine, Saint Louis, MO 63110 USA

**Keywords:** Biotechnology, Preclinical research

## Abstract

Acoustic coupling through hairs remains a challenge to performing transcranial-focused ultrasound procedures. Here, we demonstrated that this challenge could be addressed by using oil as the coupling medium, leveraging oil's high affinity to hairs due to their inherent hydrophobicity. We compared focused ultrasound-induced blood–brain barrier opening (FUS-BBBO) outcomes in mice under three coupling conditions: oil with hairs (“oil + hairs”), ultrasound gel with hair shaving (“ultrasound gel + no hair”), and ultrasound gel with hairs (“ultrasound gel + hairs”). The quality of the coupling was evaluated by ${\mathrm{T}}_{2}$-weighted magnetic resonance imaging (MRI) and passive cavitation detection (PCD). The outcome of FUS-BBBO was assessed by MRI contrast agent extravasation using in vivo ${\mathrm{T}}_{1}$-weighted contrast-enhanced MRI. It was also evaluated by ex vivo fluorescence imaging of the mouse brain after intravenous injection of a model drug, Evans blue. The results showed that “oil + hairs” consistently achieved high-quality acoustic coupling without trapping air bubbles. The FUS-BBBO outcome was not significantly different between the “oil + hairs” and the “ultrasound gel + no hair” groups. These two groups had significantly higher levels of BBB opening than the “ultrasound gel + hairs” group. This study demonstrated that oil could be a coupling medium for transcranial FUS procedures without shaving hairs.

## Introduction

Recent United States Food and Drug Administration approval of the transcranial focused ultrasound (tFUS) technique for thermal ablation treatment of essential tremors marked the beginning of a new era for incision-less neurointerventions^1^. tFUS provides a platform technology with potential clinical applications beyond thermal ablation^2^. One promising application in brain drug delivery is using low-intensity tFUS combined with microbubbles for blood–brain barrier opening (FUS-BBBO)^3^. The feasibility and safety of the FUS-BBBO technique have been demonstrated in patients with various brain diseases, including glioma, amyotrophic lateral sclerosis (ALS), Alzheimer's disease, and Parkinson's disease^4–7^. Despite the great promise of tFUS, most reported applications, except for ultrasound neuromodulation^8,9^, require the shaving of hairs to achieve efficient and consistent delivery of ultrasound energy to the brain^4,5,10,11^. Shaving hair causes significant psychological impacts on patients, including diminishing self-esteem, insecurity, and distress, especially in female patients^12^. This may lead to a lower willingness from the female patients to participate in tFUS studies^13^. The psychological impact of removing hairs could decrease the attraction of tFUS to patients and physicians and limit the scope of future tFUS clinical studies.

Only limited work has been performed to investigate the effect of hairs on ultrasound transmission, and all reported studies were performed ex vivo using human skull specimens. Raymond et al*.* demonstrated that thoroughly degassed hairs in a water tank only caused < 10% loss to transmitted ultrasound power and minimal distortion to beam shape for frequencies under 1 MHz^13^. Eames et al*.* demonstrated that thoroughly degassed hairs in a water tank caused nearly no decrease in transcranial heating efficiency when operated at 220 kHz and a 17% deduction at 710 kHz, indicating that thermal loss induced by degassed hairs was minimal^14^. These two studies suggested that tFUS treatment could be performed without shaving if hairs were fully degassed. As a result, existing tFUS procedures often require shaving hairs and then applying ultrasound gel to achieve sufficient acoustic coupling. It is worth noting that several FUS-BBBO clinical trials are currently carried out using an MRI-guided FUS system, ExAblate Neuro (Insightec, Dallas, TX) without shaving the hair. Instead, hairs are cut short and degassed in a helmet-shaped FUS transducer coupled with a water bladder. However, this requires a specially designed FUS transducer with a degassing unit, which is unavailable in other tFUS devices. Moreover, by the time this manuscript was finished, no published data on this acoustic coupling method had been published.

Ultrasound gel is a water-based liquid with high viscosity. Ultrasound gel has a superior acoustic transmission coefficient and lower reflection coefficient compared with other coupling materials, such as mineral oil, and has been widely used as the coupling layer between the scalp and the FUS transducer^15^. However, air bubbles can be easily trapped at the hair surface. These air bubbles scatter and reflect the transmitted ultrasound, significantly decreasing the intracranial ultrasound pressure and distorting the pressure distribution at the target. These air bubbles also distort microbubble cavitation signals emitted from the brain and prevent effective transcranial passive cavitation monitoring. A better coupling medium is needed to minimize the trapping of air bubbles at the hair surface and to avoid shaving hairs.

Hair is naturally covered in sebum, consisting primarily of lipids and wax produced by glands on the scalp^16^. This layer of lipids contributes to the hydrophobic nature of the hair surface. When the hydrophobic hair surface contacts water, a depletion layer may form between the water molecules and the hair surface^17^, allowing air bubbles to reside. Oil, as a hydrophobic liquid, has a higher affinity for hair surfaces than water, which has the potential to provide acoustic coupling in the presence of hairs. Mineral oil has previously been used as an acoustic coupling medium for therapeutic ultrasound applications^18,19^ but has not been used for coupling through hair.

This study aimed to evaluate the effectiveness of oil as a coupling medium for tFUS treatment without shaving hairs. To achieve this objective, we compared the outcomes of FUS-BBBO in mice under three coupling conditions: (1) oil with hairs and without ultrasound gel (“oil + hairs”), (2) ultrasound gel with hair shaving (“ultrasound gel + no hair”), and (3) ultrasound gel with hairs (“ultrasound gel + hairs”). “Oil + hairs” was performed by applying oil followed by ultrasound gel. “Ultrasound gel + no hair” was performed using the established procedure, hence the positive control group, which involves shaving the hairs and applying the ultrasound^14^. We added the third group as a negative control group, with counterpart conditions to the “oil + hairs,” to directly compare water-based and oil-based coupling solutions in the presence of hairs. Degassed water was applied to the hairs, followed by the application of the ultrasound gel. The quality of the acoustic coupling and FUS-BBBO outcome were compared among these three groups.

## Methods

### Animal preparation

All animal procedures were reviewed and approved by the Institutional Animal Care and Use Committee of Washington University in St. Louis in accordance with the National Institutes of Health Guidelines for animal research. This study is reported in accordance with ARRIVE guidelines. A total of 15 mice (Swiss, 6–8 weeks old, female, Charles River Laboratory, Wilmington, MA) were used. These mice were randomly divided into three groups to compare the outcome of FUS-BBBO under the three coupling conditions: “oil + hairs” (n = 5), “ultrasound gel + no hair” (n = 5), and “ultrasound gel + hairs” (n = 5).

### FUS-BBBO experimental setup

**Figure 1:**
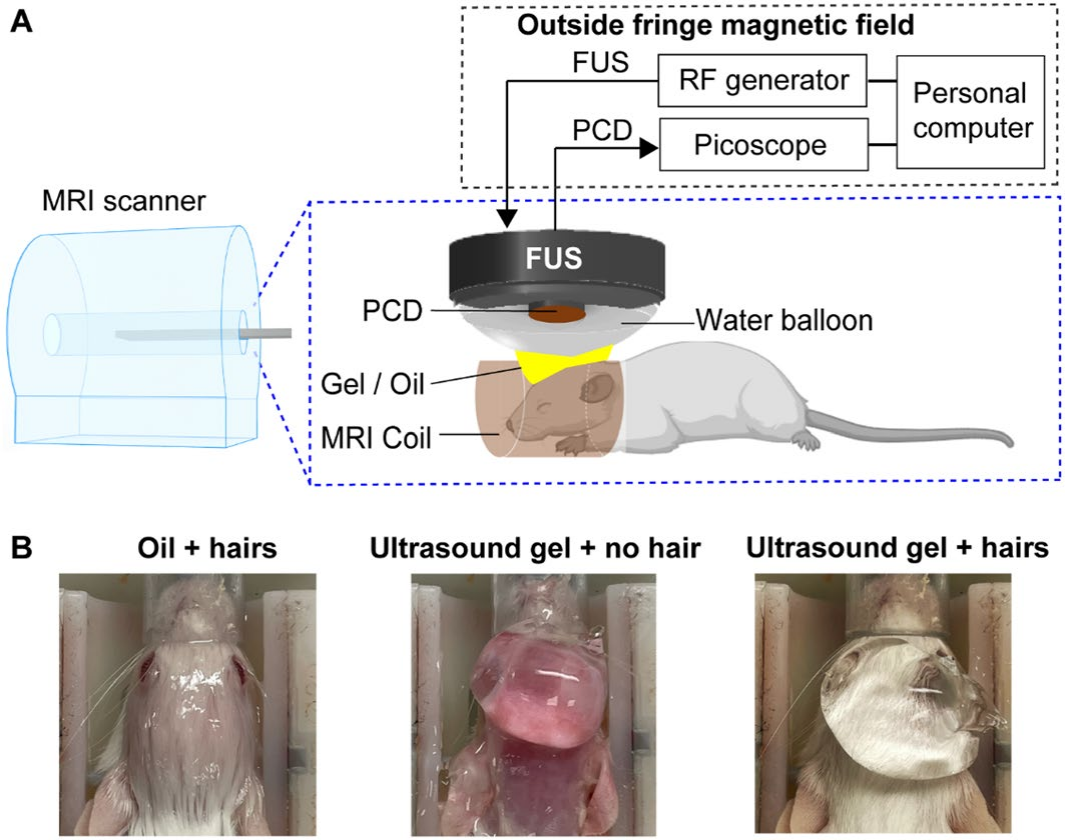
FUS-BBBO experimental setup in mice. (**A**) The experimental setup consisted of 9.4 T small animal MRI scanner and a commercial FUS system. (**B**) Different coupling methods were used for each group of mice: “oil + hairs”, “ultrasound gel + no hair”, and “ultrasound gel + hairs”.

An MR-guided FUS system (Image Guided Therapy, Pessac, France) was used to perform the FUS-BBBO procedure in mice. A schematic diagram of the experimental system is shown in Fig. 1A. This system was used in our previous MR-guided FUS-BBBO studies^10,20^. The system consisted of an MRI-compatible FUS transducer (Imasonic, Voray-sur-l'Ognon, France) made of a seven-element annular array with a center frequency of 1.5 MHz, an aperture of 25 mm, and a radius of curvature of 20 mm. The system was integrated into a 9.4 T small animal MRI scanner (Bruker, Billerica, MA, USA). The transducer was connected to an MRI-compatible piezoelectric motor, allowing the position of the transducer to be mechanically adjusted in the lateral directions (along the x- and y-axes, Fig. 1A). The axial and lateral full widths at half maximums of the FUS transducer were 5.5 mm and 1.2 mm, respectively. The acoustic pressure reported in this study was corrected for 18% mouse skull insertion loss^10,20^. A passive cavitation detection (PCD) sensor integrated at the center of the FUS transducer had a center frequency of 1.6 MHz and a − 6-dB bandwidth of 754 kHz. The signals detected with the PCD sensor were acquired via the PicoScope (5244B, Pico Technology, Cambridgeshire, UK) to monitor cavitation events. The transducer set (FUS transducer and PCD) was connected to a water balloon filled with deionized and degassed water.

### Acoustic coupling methods

The water balloon of the transducer was coupled to the mouse head using different coupling methods, as shown in Fig. 1A and B.

For the “oil + hairs” group, mineral oil (Walgreen Company, Deerfield, IL) was first poured into a weighing paper bowl and waited for at least 5 min to allow trapped bubbles, if there were any, to float and dissipate from the oil. A portion of the mineral oil was then loaded in a syringe. The rest was used to soak cotton swaps. Drops of oil were then applied onto the mouse's head using a syringe, and the oil was gently spread and brushed with oil-dipped cotton swaps. Then, 1–2 mL of oil was added on top of the hairs using a syringe. The height of the FUS transducer was then adjusted such that the transducer membrane was in contact with the oil.

For the “ultrasound gel + no hair” group, hairs were removed using Nair (Church & Dwight Co., Princeton, NJ, USA), and the scalp was thoroughly cleaned using alcohol pads. Afterward, degassed ultrasound gel (Aquasonic, Parker Laboratories, Inc., Fairfield, NJ, USA) was applied, and the FUS transducer was positioned to couple with the ultrasound gel.

For the “ultrasound gel + hairs” group, hairs were first thoroughly cleaned using alcohol pads. Degassed water was added to the hairs, and ultrasound gel was applied to the wetted hairs. The FUS transducer was then coupled with the ultrasound gel.

### FUS-BBBO procedure

Following the completion of acoustic coupling, T_2_-weighted MRI scans (TR/TE: 2200/35; slice thickness: 0.5 mm; in-plane resolution: 0.125 mm; matrix size: 256 × 256) were performed to acquire the relative location of the FUS transducer to the brain. The left striatum was chosen as the targeted brain location (FUS +), and the contralateral side was chosen as the nonsonicated control (FUS-). The transducer set (FUS transducer and PCD) was turned on, and during sonication by each FUS pulse, acoustic emission from microbubbles was recorded by the PCD. The acoustic parameters were kept the same among all three groups (0.6 MPa peak negative pressure in situ, 5 Hz pulse repetition frequency, 10,000 cycles, 3.3% duty cycle, and 3 min sonication duration). Thirty seconds after the onset of FUS sonication, commercial microbubbles (Definity, Lantheus Medical Imaging, North Billerica, MA, USA) were administered intravenously at a concentration of $8\times {10}^{8}$ bubbles/mL and a total volume of 30 µL, followed by a saline flush. Immediately after FUS treatment, 4% Evans blue was delivered intravenously as a model drug.

### PCD signal processing

Similar to our past publication^10^, a custom MATLAB script was written to process the acquired PCD data for the evaluation of stable cavitation doses. Briefly, baseline PCD data was acquired during the initial 30 s sonication before microbubble injection. After microbubbles were injected, PCD data were acquired until the sonication ended. The stable cavitation dose was calculated with the following steps: (1) The mean of the pre-microbubble injection stable cavitation (SC) level was calculated by averaging the SC levels observed before microbubble injection. The SC level was assessed in the frequency domain by summing the amplitude at the second harmonic (3.0 MHz) within a ± 0.02 MHz bandwidth. (2) The post-microbubble injection SC level was determined by subtracting the mean of the pre-microbubble SC level from the SC level calculated at each time point. (3) The stable cavitation dose was then quantified by summing the SC levels between the time after microbubble injection and the end of sonication. These steps ensured the consideration of the variations in the baseline SC level for individual mice.

### MRI evaluation of the acoustic coupling quality

T_2_-weighted MRI scan (TR/TE, 4228/35; slice thickness: 0.5 mm; in-plane resolution: 0.125 mm; matrix size: 256 × 256) was performed at the top of the mouse head to evaluate the quality of the acoustic coupling. As a quantitative assessment, we calculated the number of air bubbles trapped in the coupling medium within a region of interest (ROI) at the interface between the coupling medium and the hair/skin of the mice. The size of the ROI (40 × 24 pixels, pixel width of 0.125 mm) was kept consistent throughout all objects. The location of ROI was fixed on the posterior portion of the skin/hair-coupling medium interface region, which appeared as the gray area in the T_2_-weighted MRI images. The air bubbles inside the ROI were then extracted by post-processing using ImageJ^21^.

### FUS-BBBO outcome assessment

The FUS-BBBO outcome was assessed in vivo using contrast-enhanced T_1_-weighted MRI and ex vivo by fluorescence imaging of brain slices.

In vivo*,* contrast-enhanced T_1_-weighted MRI scan (TR/TE: 20/5; slice thickness: 0.13 mm; in-plane resolution: 0.13 mm; matrix size: 120 × 240; flip angle: 20°) was performed to evaluate the outcome of FUS-BBBO based on the extravasation of the MRI contrast agent gadobenate dimeglumin (Gd-BOPTA; MultiHance, Bracco Diagnostics Inc., Monrow Township, NJ) from the blood circulation into brain tissue. BBBO volume was calculated by comparing the contrast-enhanced volume in the T_1_-weighted images on the FUS + and FUS- sides using a custom MATLAB script reported in our previous publications^10,20,22^. Briefly, the contrast-enhanced volume for each mouse was calculated by the sum of voxels in the FUS + side with an intensity above the mean plus three times the standard deviation of the FUS- side for each individual scan slice.

All mice were sacrificed under vaporized isoflurane anesthesia at around 30 min after FUS sonication by transcardial perfusion with 30 mL 1 × PBS for 5 min. Brains were harvested and fixed in 4% paraformaldehyde for at least 24 h. The brains were then transversely cut into 1 mm slices using a brain matrix (RBM-4000C, ASI Instruments Inc., MI, USA) and imaged using the Pearl Trilogy Image System (LI-COR, Lincoln, NE, USA). Evans blue delivery outcome was quantified using the system's built-in software (Image Studio Lite, LI-COR, Lincoln, NE, USA). The fluorescence intensity within the FUS + side was summed and normalized by the sum of the fluorescence intensity of the contralateral side of the striatum for each individual brain slice.

### Statistical analysis

Statistical analyses were performed using GraphPad Prism (Version 9.0, La Jolla, CA, USA). Differences among multiple groups were determined using ordinary one-way ANOVA with group-wise comparisons. A *p* value < 0.05 was used to determine statistical significance.

## Results

**Figure 2:**
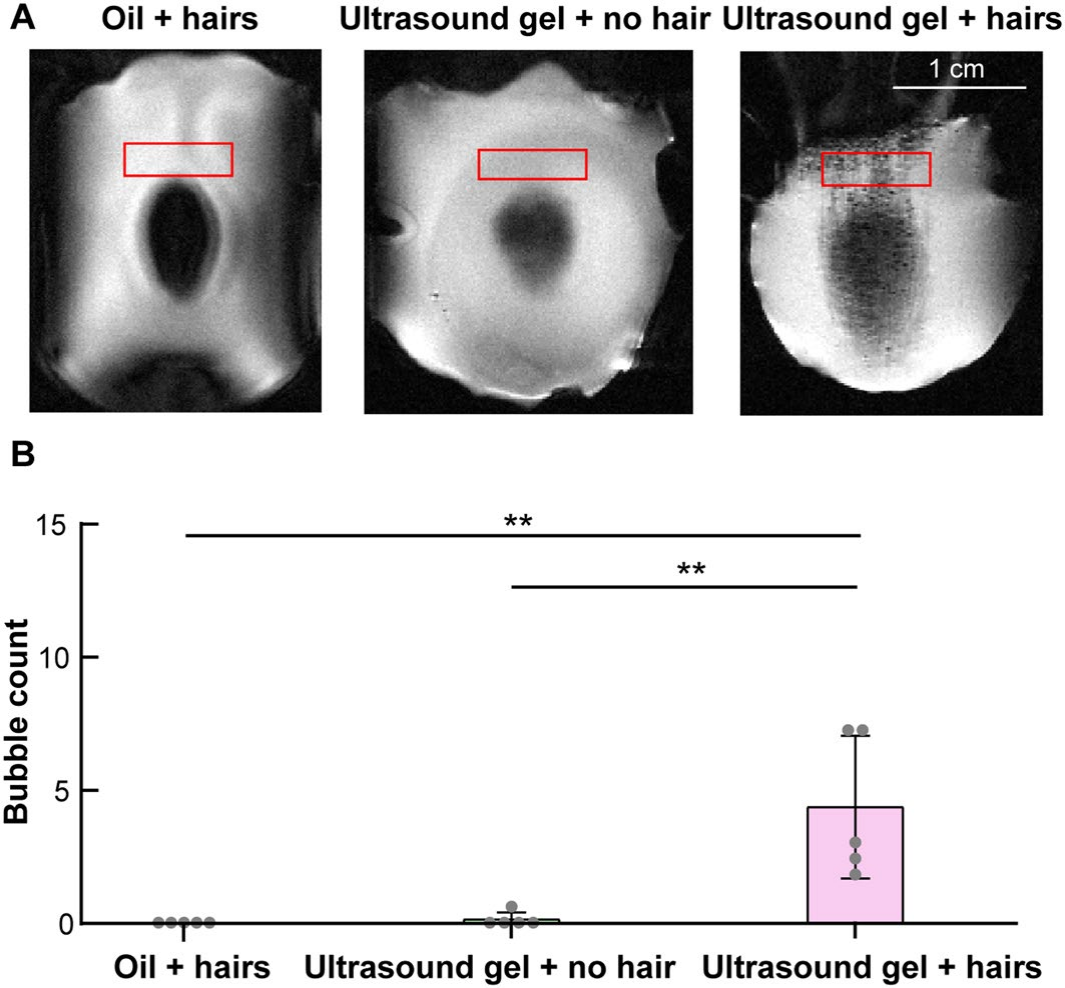
“Oil + hairs” achieved clean acoustic coupling without bubbles. (**A**) Representative transversal ${\mathrm{T}}_{2}$-weighted MRI images of the scalp for all three groups. More bubbles, as shown by black dots, are visible in ${\mathrm{T}}_{2}$-weighted images for the “ultrasound gel + hairs” group than for the “oil + hairs” and “ultrasound gel + no hair” groups. The region of interest (ROI) for the quantification of bubble numbers is highlighted by the red box in each image. (B) Quantification of the number of air bubbles trapped in the coupling medium within the ROI for each group of mice. **p < 0.01.

${\mathrm{T}}_{2}$-weighted MRI showed that “oil + hairs” and “ultrasound gel + no hair” achieved similar bubble-free coupling, while the “ultrasound gel + hairs” group had bubbles trapped in the coupling medium (Fig. 2). Quantification of bubble numbers within the selected ROI found that there were significantly more bubbles in the acoustic coupling medium in the “ultrasound gel + hairs” group compared with the “oil + hairs” and “ultrasound gel + no hair” groups (*p* = 0.0022 and 0.0028, respectively). No significant difference in bubble count was found between the “ultrasound gel + no hair” and “oil + hairs” groups.

**Figure 3:**
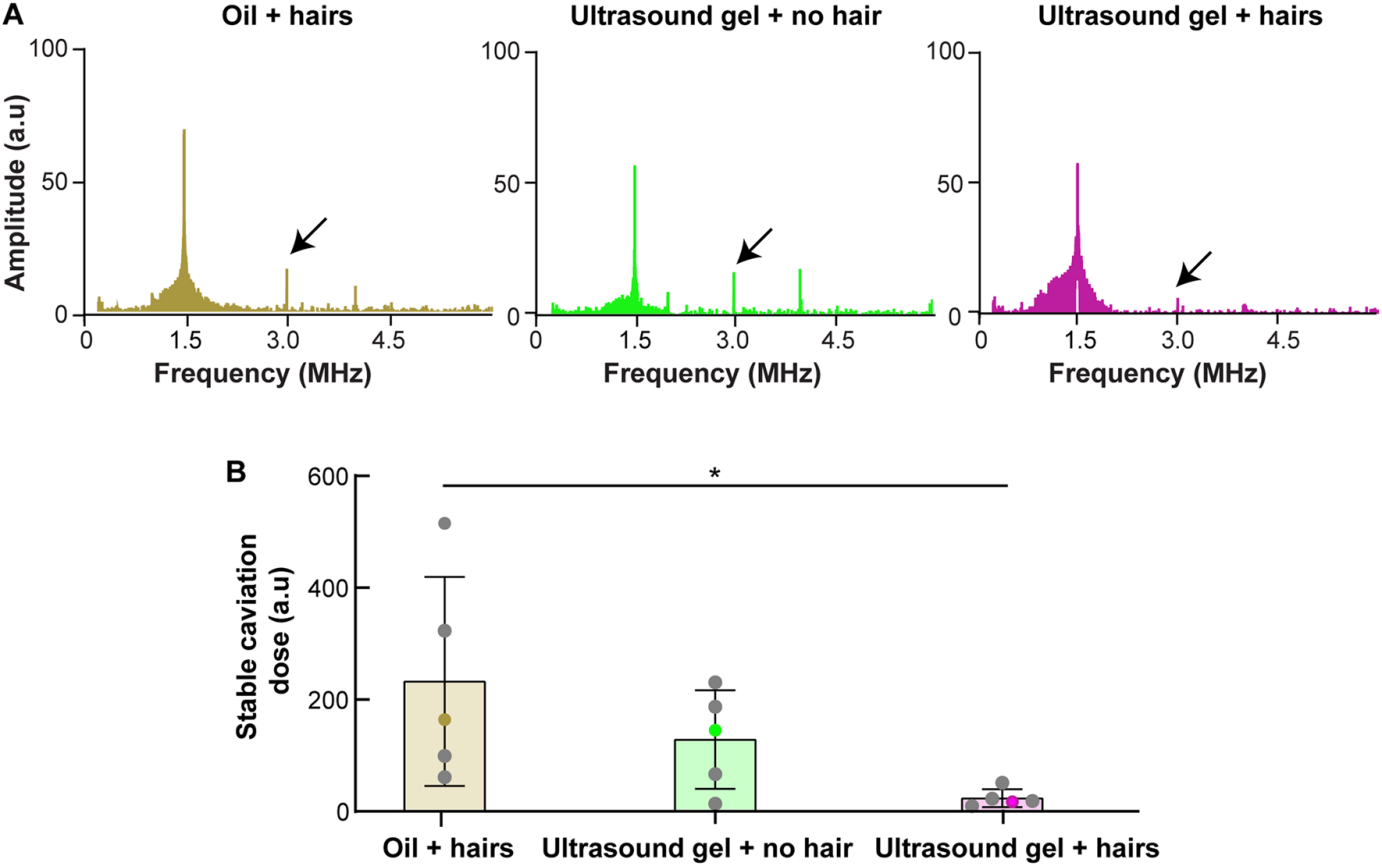
“Oil + hairs” achieved a stable cavitation dose comparable to that of the conventional “ultrasound gel + hairs.” (**A**) Representative single-pulsed PCD spectral signals for “oil + hairs,” “ultrasound gel + no hair,” and “ultrasound gel + hairs” for one mouse. The second harmonic signal, which was used as a marker for stable cavitation, is indicated by an arrow in each plot. (**B**) Quantification of the stable cavitation doses for the three groups. A significant difference was observed between the “oil + hairs” and “ultrasound gel + hairs” groups (*p* = 0.0428). Data calculated for the representative subjects shown in A are highlighted with their corresponding color (brown for “Oil + hairs,” green for “Ultrasound gel + no hair,” and purple for “Ultrasound gel + hairs”). *p < 0.05.

Figure 3A presents each group's representative frequency spectrum of the acquired PCD signal. A higher spectrum amplitude was observed for the “oil + hairs” and “ultrasound gel + no hair” groups than for the “ultrasound gel + hairs” group at the second harmonic frequency. Figure 3B shows no significant difference in the stable cavitation dose was observed between the “oil + hairs” and “ultrasound gel + no hair” groups. The stable cavitation dose for the “oil + hairs” group was significantly higher than that for the “ultrasound gel + hairs” group (*p* = 0.0428).

**Figure 4:**
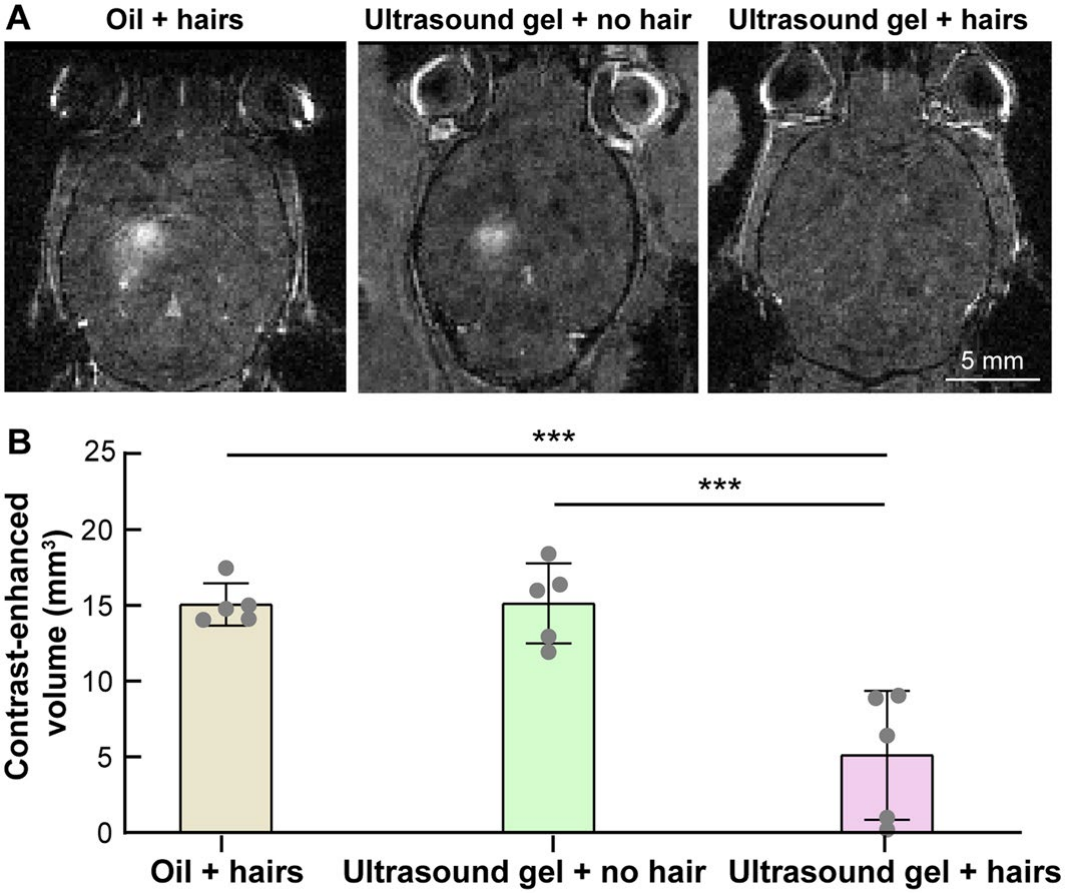
“Oil + hairs” achieved comparable BBBO volume as the conventional “ultrasound gel + hairs.” (**A**) Representative contrast-enhanced ${\mathrm{T}}_{1}$-weighted MRI images for “oil + hairs”, “ultrasound gel + no hair”, and “ultrasound gel + hairs”. (**B**) The contrast-enhanced volume was compared among these three groups. The contrast-enhanced volumes of the “oil + hairs” group (*p* = 0.0002) and the “ultrasound gel + no hair” group (*p* = 0.0002) were significantly higher than that of the “ultrasound gel + hairs” group. ***p < 0.001.

Successful BBB opening was achieved in five out of five mice in the “oil + hairs” group and “ultrasound gel + no hairs” group (Fig. 4A). Three out of five mice in the “ultrasound gel + hairs” group also had low levels of BBB opening. The contrast-enhanced volume, as measured based on contrast-enhanced MRI, was not significantly different between the “oil + hairs” group (15.1 ± 1.4 mm^3^) and the “ultrasound gel + no hair” group (14.6 ± 2.4 mm^3^). Both groups achieved significantly higher contrast enhancement volumes than the “ultrasound gel + hairs” group (*p* = 0.0002 and *p* = 0.0002, respectively) (Fig. 4B).

**Figure 5:**
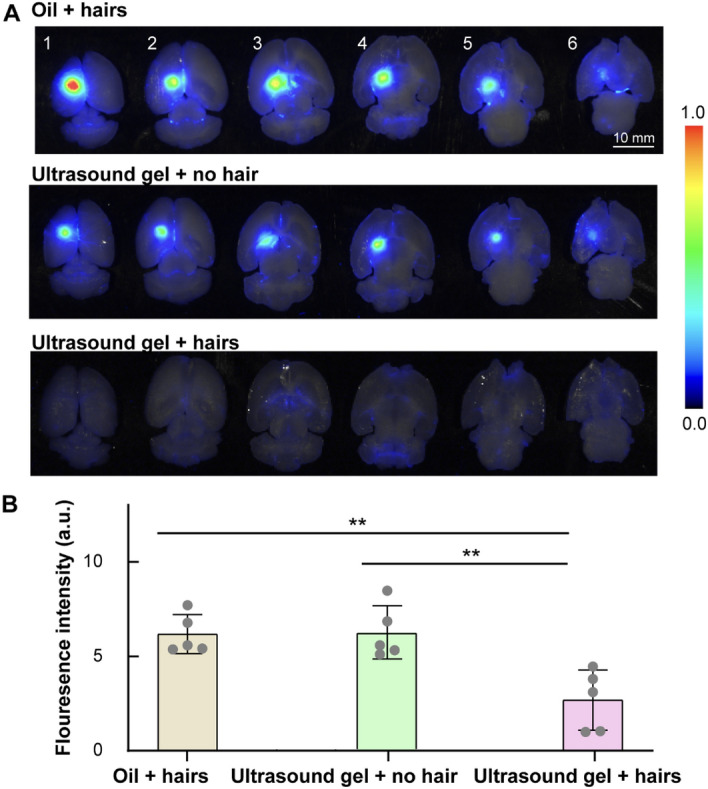
“Oil + hairs” achieved comparable drug delivery as the conventional “ultrasound gel + hairs”. (**A**) Ex vivo fluorescence images of Evans blue delivery in all five mouse brains in the three groups. (**B**) Quantification of the Evans blue fluorescence intensity for the three groups. The fluorescence intensity was significantly higher for the “oil + hairs” (*p* = 0.0043) and “ultrasound gel + no hair” (*p* = 0.0035) groups than for the “ultrasound gel + hairs” group. **p< 0.01.

Consistently, the Evans blue intensity was not significantly different between the “oil + hairs” group and the “ultrasound gel + no hair” group. Both groups had significantly higher Evans blue delivery than the “ultrasound gel + hairs” group (*p* = 0.0043 and *p* = 0.0035, respectively) (Fig. 5B).

## Discussion

This study presented a practical solution for acoustic coupling without shaving hairs. It used oil as a coupling medium instead of water-based ultrasound gel. The FUS-BBBO outcome utilizing the oil as a coupling medium without shaving hairs was not significantly different from that achieved using the conventional approach, which required shaving hairs and then applying the ultrasound gel. This study demonstrated for the first time that in vivo FUS-BBBO can be done without shaving or shortening hairs.

The hydrophobic nature of oil is advantageous over conventional water-based ultrasound gels for acoustic coupling in the presence of hairs. Glands on scalps on animals and humans naturally secrete sebum, a mixture of lipids and wax, which forms a hydrophobic surface on hairs. This surface impedes the bonding between water molecules and hair cuticles, making it challenging for water and water-based gel to fill in the space between different layers of hair cuticles and ultimately contributes to the trapping of air bubbles when applying the ultrasound gel^17^. More small bubbles were observed in the T_2_-weighted images (Fig. 2) in mice treated with ultrasound gel on hairs than in those treated with oil on hairs. Furthermore, oil can be conveniently applied to patients without causing any irritation to the skin. It can be easily cleaned with shampoo or detergent after the procedure is finished, making it an excellent solution for broad applications in tFUS therapies. It is worth noting that we used mineral oil in this study because it is widely available. Still, we expect that a wide range of oil- and lipid-based solutions can be used to achieve similar outcomes, as demonstrated in this study.

The “oil + hairs” coupling method is easy to implement and as effective as the conventional “ultrasound gel + no hair” method. Cavitation monitoring by PCD (Fig. 3), BBBO volume quantification by in vivo contrast-enhanced MRI (Fig. 4), and drug delivery efficiency evaluation by ex vivo fluorescence imaging (Fig. 5) found no significant difference between “oil + hairs” and the conventional “ultrasound gel + no hair.” This finding suggests that the current coupling method can be replaced with oil without compromising the BBBO outcome.

Although we achieved satisfactory FUS-BBBO outcomes through hairs using oil as a coupling medium, our study has a few limitations. Mouse hairs are different from human hairs, making it much easier for oil to stay. The head shape of mice is relatively flat compared with that of humans. Oil may be harder to stay on the human head than on the mouse. These limitations can potentially be mitigated by using a more viscous oil solution. Future studies are warranted to evaluate and optimize this acoustic coupling approach for humans. Also, other hydrophobic solutions such as hydrophobic hydrogel and petroleum gel can be explored as alternative coupling medium for mineral oil in future studies.

## Conclusions

Using oil instead of ultrasound gel as the coupling medium was proposed to address the critical need for a practical solution to achieve high-quality acoustic coupling without shaving hairs. Oil was used as an alternative coupling medium instead of water-based ultrasound gel, leveraging the hydrophobicity of the hair's surface. Three coupling methods (“oil + hairs”, “ultrasound gel + no hair”, and “ultrasound gel + hairs”) were compared. The results showed that applying oil directly on hairs achieved comparable FUS-BBBO outcomes compared with the conventional method, which applied ultrasound gel after shaving hairs. This proof-of-concept study laid the foundation for the future development of tFUS techniques without shaving hairs.

## Data Availability

The datasets used and analyzed during the current study are available from the corresponding author.
